# Antioxidant and Anti-Inflammatory Effects of Selected Natural Compounds Contained in a Dietary Supplement on Two Human Immortalized Keratinocyte Lines

**DOI:** 10.1155/2014/327452

**Published:** 2014-08-17

**Authors:** Elena Fasano, Simona Serini, Nadia Mondella, Sonia Trombino, Leonardo Celleno, Paola Lanza, Achille Cittadini, Gabriella Calviello

**Affiliations:** ^1^Institute of General Pathology, Catholic University, 00168 Rome, Italy; ^2^Department of Pharmacy, Health and Nutritional Sciences, University of Calabria, Arcavacata di Rende, 87030 Cosenza, Italy; ^3^Institute of Clinical Dermatology, Catholic University, 00168 Rome, Italy; ^4^Research Center for Biotechnology Applied to Cosmetology, Catholic University, 00168 Rome, Italy; ^5^Institute of Pathology, Catholic University, 00168 Rome, Italy

## Abstract

Several advantages may derive from the use of dietary supplements containing multiple natural antioxidants and/or anti-inflammatory agents. At present, however, there is scarce information on the properties and potential of combined supplements. To fill the gap, the antioxidant and anti-inflammatory activities exerted by a combination of seven natural components (coenzyme Q10, krill oil, lipoic acid, resveratrol, grape seed oil, *α*-tocopherol, and selenium) contained in a dietary supplement used for the prevention of skin disorders were investigated *in vitro*. Each component was administered, alone or in combination, to human keratinocytes, and the inhibition of Reactive Oxygen Species production and lipid peroxidation as well as the ability to reduce inflammatory cytokine secretion and to modulate Nuclear Factor-*κ*B pathway was evaluated. The combination exhibited high antioxidant activity and in specific conditions the combination's efficiency was higher than that of the most powerful components administered individually. Moreover, the combination showed remarkable anti-inflammatory properties. It reduced more efficiently than each component the secretion of Monocyte Chemoattractant Protein-1, a crucial cytokine for the development of chronic inflammation in skin, and inhibited Nuclear Factor-*κ*B molecular pathway. Overall, our findings suggest that the combined formulation may have the potential to powerfully inhibit oxidative stress and inflammation at skin level.

## 1. Introduction

The beneficial effects of natural compounds with antioxidant and anti-inflammatory activities in the prevention of different chronic pathologies have been studied for many years in the laboratory where the present study was conducted [1–3]. Actually, the benefit resulting from a dietary supplementation with these bioactive compounds once isolated from natural sources is a quite controversial issue. Whereas most preclinical and observational human studies demonstrated the ability of these supplements to reduce the risk of several chronic diseases, contrasting outcomes were obtained in intervention human studies, and sometimes their use was associated with an increased disease risk [4]. However, these compounds are often supplemented at extremely high doses, either in combination or individually, in capsules, pills, tablets, powders, drinks, and so forth, and in these forms they may either induce prooxidant and cytotoxic effects [5] or become extremely unstable. Conversely, it is known that the natural combination of different intracellular antioxidants (both enzymatic and nonenzymatic) ensures the reciprocal protection against oxidative damage and instability and induces synergic and interdependent effects [6]. Even though a well-balanced diet may provide an adequate combination of antioxidants [4] and prevent pathologies associated to oxidative stress, the dietary supplementation with formulations combining several compounds with known antioxidant/anti-inflammatory effects may represent an interesting and alternative strategy for disease prevention, especially in those regions where vegetables and other dietary sources are not easily available at reasonable prices or in acceptable amounts to satisfy the requirements of the general population. Moreover, these combined formulations could be very useful in those subjects that, due to health problems, either cannot ingest/adsorb foods at high content of antioxidants or show a high “oxidative status” [7], like sport-people or individuals predisposed to chronic diseases pathogenically related to oxidative stress and inflammation.

It was previously shown by us that synthetic molecules composed of two antioxidant substances molecularly conjugated exerted higher antioxidant effect than the parental compounds [2, 8]. Some of these antioxidant substances were also included by us in lipid nanoparticles, whose basal structure was constituted by another antioxidant compound, with the aim to transport and protect them [9]. Dietary supplements containing combinations of natural antioxidant/anti-inflammatory compounds can be presently found in the market. They are largely publicized for their powerful healthy effects, even though the available information on their properties is quite scarce [10], being dietary supplements not required to go through testing like drugs. Thus, it appears crucial to understand if such combinations are balanced, efficacious, and more powerful in preventing oxidation/inflammation than each of their bioactive components. To this aim, in the present work, the effect* in vitro* of one of these dietary supplements composed of a combination of seven components with known antioxidant and/or anti-inflammatory properties (coenzyme Q10 (CQ10), krill oil (KO), lipoic acid (LA), resveratrol (Resv), grape seed oil (GO), *α*-tocopherol (*α*-T), and Na-selenite (Se)) was studied. This dietary supplement is recommended for the prevention of skin disorders that are pathogenically related to both oxidative stress and inflammation. Each single component was administered alone or in different combinations to two human immortalized keratinocyte lines (NCTC 2544 and HaCaT) showing different degrees of differentiation [11, 12], and the antioxidant activity and the ability to inhibit the inflammatory response were evaluated. Overall, the findings obtained suggest that the combined treatment has the potential to provide additional benefits in the prevention of chronic diseases showing an oxidative and/or inflammatory pathogenesis. The possibility of a topical use of a formulation containing the combination of the compounds/oils examined has been also suggested.

## 2. Materials and Methods

### 2.1. Cell Lines

HaCaT human immortalized keratinocytes were obtained from American Type Culture Collection (ATCC, Rockville, MD, USA), and NCTC 2544 human immortalized keratinocytes were kindly gifted by Dr. R. De Bellis (Università degli Studi di Urbino, Urbino, Italy). As previously demonstrated [11, 12], these two cell lines show a different degree of differentiation and express cytokine patterns typical of either less differentiated basal layers of epidermis (NCTC 2544) or more differentiated superficial layers of epidermis (HaCaT). Both cell lines were cultured in DMEM medium containing glutamine (2 mM), antibiotics (100 U/mL penicillin and 100 *μ*g/mL streptomycin), and 10% FBS in a humidified atmosphere at 37°C and 5% CO_2_. Cells were maintained in an exponential growth rate by subculturing them at the density of 3 × 10^5^ cells/mL twice a week. Cell viability was analyzed through the Trypan blue dye exclusion method.

### 2.2. Supplement Components and Concentrations Used

The antioxidant and anti-inflammatory activities of the following compounds/oils included in the formulation of a dietary supplement were evaluated: coenzyme Q10, L.C.M. Trading SpA, Milan, Italy; krill oil, Aker Biomarine Antarctic AS, Oslo, Norway; lipoic acid, Giellepi SpA, Seregno, Milan, Italy; resveratrol, K.-W. Pfannenschmidt GMBH, Hamburg, Germany; grape seed oil, G. Balestrini Srl, Milan, Italy; *α*-tocopherol, Van Eeghein Functional Ingredients, Amsterdam, Netherlands; and Na-selenite, Giusto Faravelli SpA, Milan, Italy. These compounds/oils were administered alone or in combination to HaCaT and NCTC 2544 keratinocytes. Since the present study was performed* in vitro* on human keratinocyte lines with the aim to understand the potential activities of the dietary supplement once consumed* in vivo*, we did not use the components in the original concentrations and ratios of the formulation of the supplement. Instead, we referred to the concentrations of these components found in human plasma following their dietary consumption [13–18]. On the basis of these plasmatic concentrations, most of the authors that previously evaluated* in vitro* the beneficial effects of such components on keratinocytes or other cells used the ranges of concentrations that are reported in Table 1 [19–38]. Since these components, used within these ranges of concentration, had always showed lack of toxicity and ability to efficiently decrease oxidative stress and/or inflammation* in vitro*, in the present work concentrations within these ranges were used. In particular, three concentrations were used for all the components, according to the increasing quantitative scheme: 1, 1 × 5, and 5 × 5. The concentrations used are as follows: 1.2, 6.0, and 30.0 *μ*M for *α*-T; 0.4, 2.0, and 10.0 *μ*M for CQ10, LA, and Resv; 0.8, 4.0, and 20.0 *μ*g/mL for GO and KO. Only for Se the concentration scheme used was as follows: 1, 1 × 2.5, and 1 × 5, which corresponded to 0.08, 0.2, and 0.4 *μ*M, in order to avoid the cytotoxicity showed in preliminary experiments by Se concentrations higher than 0.4 *μ*M. In agreement, Hazane-Puch et al. [31] recently reported that concentrations higher than 1.0 *μ*M of Na-selenite exerted a cytotoxic effect on HaCaT cells. GO is not reported in Table 1 since, unlike similar oils extracted from fish or krill, it has never been administered* in vitro* to cell cultures. So far, instead, only aqueous extracts from grape seed have been used* in vitro*. On this basis, in the present work, GO was used at the same concentrations used for KO. A cell viability ≥95% was observed in preliminary experiments carried out on HaCaT and NCTC 2544 cells using all the concentrations reported above.

### 2.3. Immunocytochemical Analysis of Cytokeratin-13 (CK-13)

HaCaT and NCTC 2544 cells were trypsinized, resuspended at the density of 8 × 10^4^ cells/100 *μ*L, and cytocentrifuged by Cytospin (Shandon). “Superfrost” polarized slides were used. Slides were air-dried for 30 min; then cells were fixed in 4% paraformaldehyde for 7 min and permeabilized in Triton-X for further 7 min. To avoid the unspecific background due to endogenous peroxidases, slides were exposed to 3% H_2_O_2_ for 2 min. To inhibit potential endogenous biotin, the Avidin/Biotin kit (Vector) was used. The slides were then incubated for 30 min at room temperature with the cytokeratin-13 antibody (CK-13, clone 1C7, sc-58721, Santa Cruz Biotechnology) diluted 1 : 50 in primary Antibody Diluent (Phosphate Green, Scytek Laboratories). Slides were then incubated with the secondary antibody (10 min at room temperature) using the Ultra-Tek-HRP kit (Antipolyvalent, Scytek Laboratories).

### 2.4. Reactive Oxygen Species (ROS) Production

HaCaT and NCTC 2544 cells were seeded in 6-well multiwell plates at the density of 5 × 10^5^ cells/well. After 24 h, culture medium was removed and replaced with fresh culture medium containing or not the different compounds at increasing concentrations. After 24 h of treatment, cells were washed in phosphate buffered saline (PBS) and cell culture medium was replaced with PBS and incubated at 37°C in the dark in the presence of the fluorogenic substrate 6-carboxy-2′,7′-dihydrodichlorofluorescein diacetate (DCF, 10 *μ*M). Fluorescence was measured by a plate cytofluorometer (Cytofluor 2300/2350 Fluorescence Measurement System (Millipore Corp., Bedford, MA)) with an excitation wavelength of 485 nm and an emission wavelength of 530 nm. As a prooxidant stimulus, 100 *μ*M H_2_O_2_ was added to the wells and plates were incubated at 37°C for further 15 min and fluorescence produced was measured again. This concentration was used since it was observed in preliminary experiments that it induced the maximal prooxidant effect without causing cell necrosis (data not shown).

### 2.5. Lipid Peroxidation Analysis

HaCaT and NCTC 2544 keratinocytes were seeded in Petri dishes at the density of 3 × 10^5^ cells/mL. After 24 h, culture medium was removed and replaced with fresh culture medium containing or not the different compounds given alone or in combination. The ability of the different compounds to inhibit lipid peroxidation was analyzed both in basal conditions and in the presence of two different free radical sources: 2,2′-azobis (2-amidinopropane) (AAPH, 25 mM), which exogenously produces peroxyl radicals by thermal decomposition, and* tert*-butyl-hydroperoxide (*t*-BOOH, 0.25 mM), which endogenously produces alkoxyl radicals through Fenton reaction [3]. After 22 h of treatment, AAPH or* t*-BOOH or vehicle alone was added to culture dishes for further 2 h. Cells were then trypsinized and centrifuged and 5 × 10^6^ cells for each sample were resuspended in 200 *μ*L of PBS. Then, 200 *μ*L of 15% trichloroacetic acid (TCA) in 0.25 M HCl and 200 *μ*L of 0.37% thiobarbituric acid (TBA) in 0.25 M HCl were added. Samples were incubated at 90°C for 20 min and then centrifuged (5000 rpm for 5 min) in order to remove cell debris. The final product of lipid peroxidation is malondialdehyde (MDA) which reacts with TBA to form a coloured product measured spectrophotometrically at 535 nm (*ελ* = 1.56 ×10^5^).

### 2.6. Monocyte Chemoattractant Protein-1 (MCP-1) and Interleukin-6 (IL-6) Analysis

HaCaT cells were seeded at the density of 5 × 10^3^ cells/well in 96-well multiwell culture plates. When cells reached confluence, culture medium was removed and replaced with fresh culture medium containing the different compounds in the presence or not of the proinflammatory cytokine tumor necrosis factor-*α* (TNF-*α*, 20 ng/mL). After a 24 h treatment (6 h pretreatment with TNF-*α* and further 18 h treatment with the compounds), supernatant was collected, centrifuged to remove suspended cells, and analyzed by immunoenzymatic assay utilizing commercial ELISA kits (Biolegend, San Diego, CA, USA). The minimum detectable concentrations of MCP-1 and IL-6 were 3.9 pg/mL and 4.0 pg/mL, respectively.

### 2.7. Analysis of p65, I*κ*B*α*, and p-I*κ*B*α*

Protein expression was evaluated by Western Blotting analysis. Briefly, after the indicated treatments, HaCaT cells were trypsinized, centrifuged, and lysed for 30 min in ice-cold lysis buffer (150 mM NaCl, 1.0% NP-40, 0.5% sodium deoxycholate, 0.1% sodium dodecyl sulphate (SDS), and 50 mM Tris, pH 8.0) containing protease and phosphatase inhibitors (50 KIU/mL aprotinin, 5 *μ*g/mL leupeptin, 100 *μ*M phenylmethanesulfonyl fluoride (PMSF), 1 mM Na_3_VO_4_, 10 mM NaF, 1 *μ*M pepstatin, and 100 *μ*M dithiothreitol (DTT)). Samples were then centrifuged at 14000 rpm for 10 min and supernatants were collected. Protein concentration in samples was measured through Bio-Rad method. 80 *μ*g of total proteins was separated in a 10% acrylamide/bisacrylamide gel and then transferred on a polyvinylidene difluoride (PVDF) membrane. The membrane was blocked in nonfat dry milk (5% in PBS containing 0.05% Tween (TBST) 1X or in bovine serum albumin (BSA), 3% in TBST 1X, for the expression of p-I*κ*B*α*) for 1 h at room temperature and incubated overnight at 4°C in the presence of primary antibodies against p65 [(C-20): sc-372], I*κ*B*α* [(H-4): sc-1643], and p-I*κ*B*α* [(B-9): sc-8404] (Santa Cruz Biotechnology, CA, USA). As a loading control, membranes were reincubated in the presence of a primary antibody against *α*-actinin [(H-2): sc-17829] (Santa Cruz Biotechnology, CA, USA). Membranes were then washed in TBST 1X and incubated in the presence of anti-mouse (1 : 10000, for I*κ*B*α*, p-I*κ*B*α*, and *α*-actinin) or anti-rabbit (1 : 20000, for p65) secondary antibodies in TBST 1X for 1 h at room temperature. Proteins were visualized incubating the membranes in the reagents for chemiluminescence detection. Protein quantification was performed by densitometric analysis.

### 2.8. Statistical Analysis

**Figure 1:**
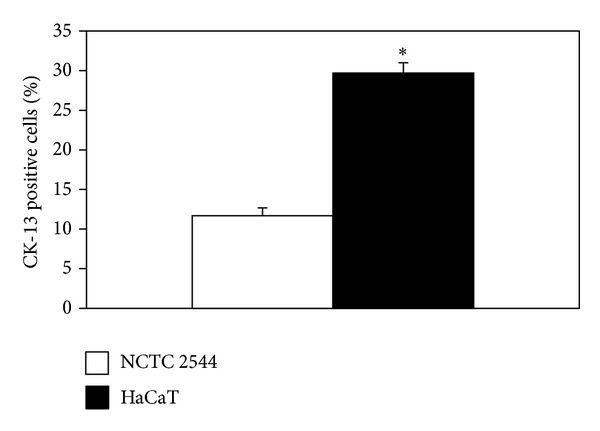
Cytokeratin-13 (CK-13) basal expression in NCTC 2544 and HaCaT cells. CK-13-positive cells were evaluated by immunocytochemistry and counted under inverted microscopy. Values represent the means ± SE of CK-13-positive cells counted in 5 and 8 microscopic fields for NCTC 2544 and HaCaT cells, respectively. ∗: significantly different, *P* < 0.0001, two-tailed unpaired *t*-test.

**Figure 2:**
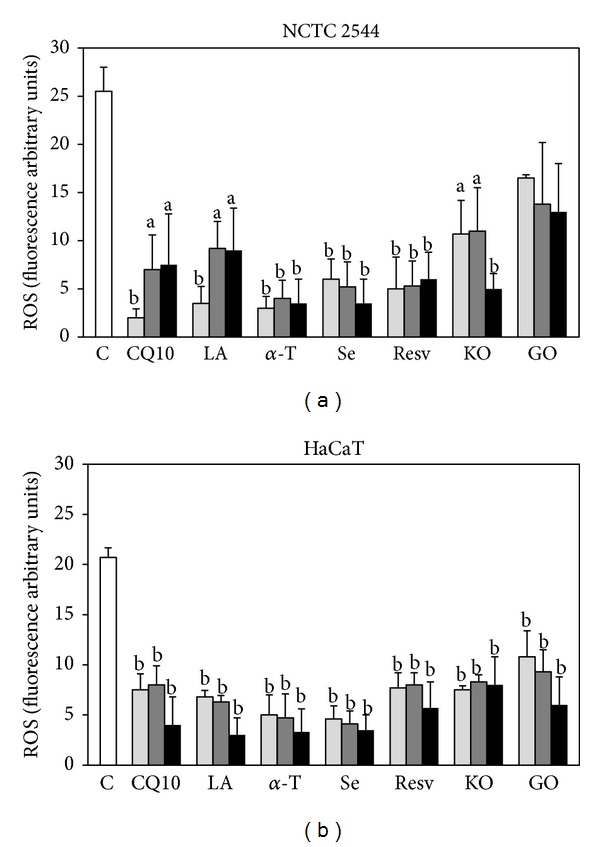
Effect of the seven supplement's components on ROS production in keratinocytes in basal conditions. Cells were treated for 24 h with three increasing concentrations of the components administered individually (see Section 2). Data are the means ± SE of a number of determinations ranging from 3 to 8. a: significantly different from control (*P* < 0.05); b: significantly different from control (*P* < 0.01), one-way ANOVA followed by Dunnett's test.

**Figure 3:**
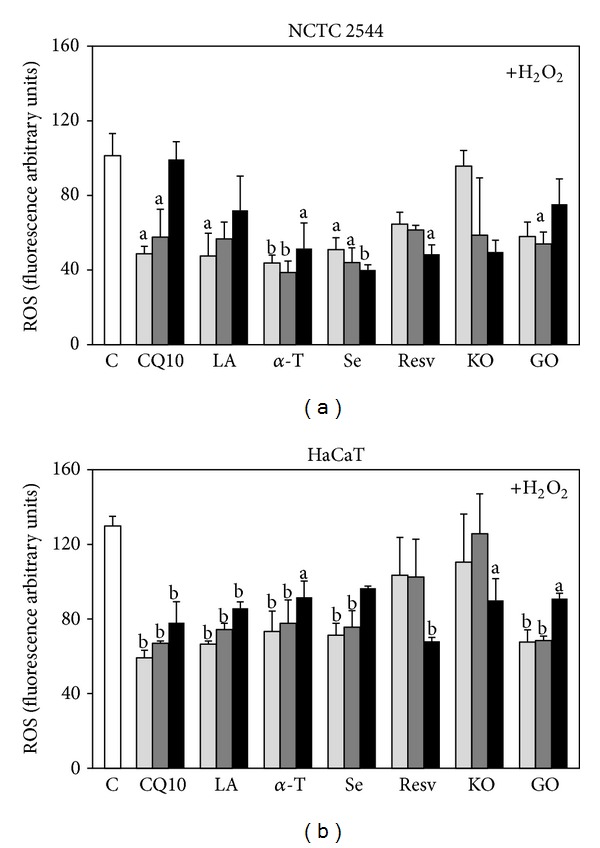
Effect of the seven supplement's components on ROS production in keratinocytes exposed to H_2_O_2_. Cells were treated for 24 h with three increasing concentrations of the components and then exposed to 100 *μ*M H_2_O_2_ for 30 min. The three concentrations used for each component are reported in Section 2. Data are the means ± SE of a number of determinations ranging from 3 to 8. a: significantly different from control (*P* < 0.05); b: significantly different from control (*P* < 0.01), one-way ANOVA followed by Dunnett's test.

**Figure 4:**
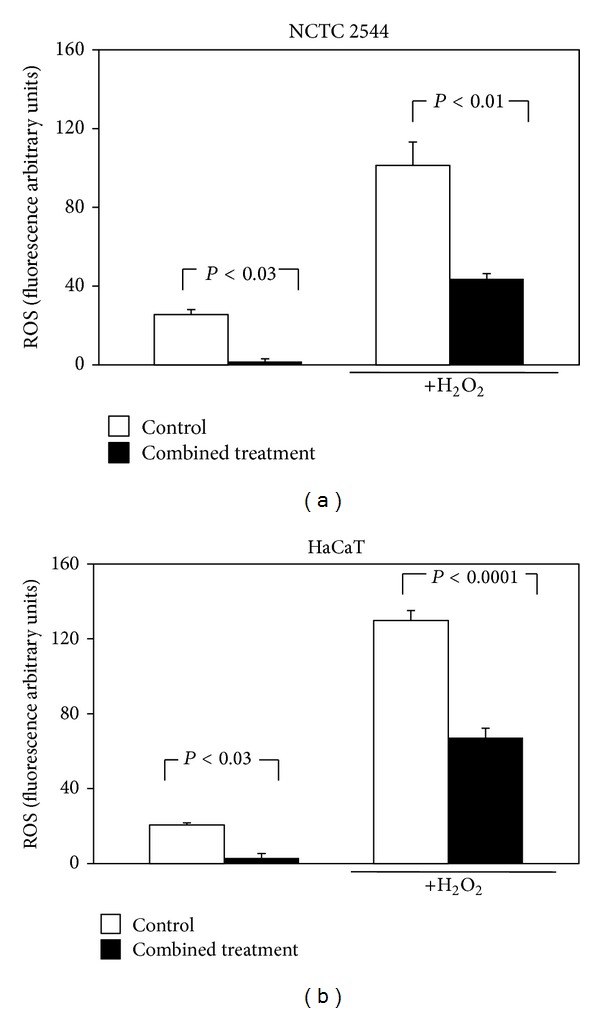
Effect of the combined treatment with all the seven supplement's components on ROS production in keratinocytes exposed or not to H_2_O_2_. Cells were treated simultaneously for 24 h with all the components and then exposed to H_2_O_2_ (100 *μ*M, 30 min) or vehicle alone. Each component was used at the highest concentration reported in Section 2. Data are the means ± SE of a number of determinations ranging from 3 to 8. Significance was evaluated by the two-tailed unpaired *t*-test.

**Figure 5:**
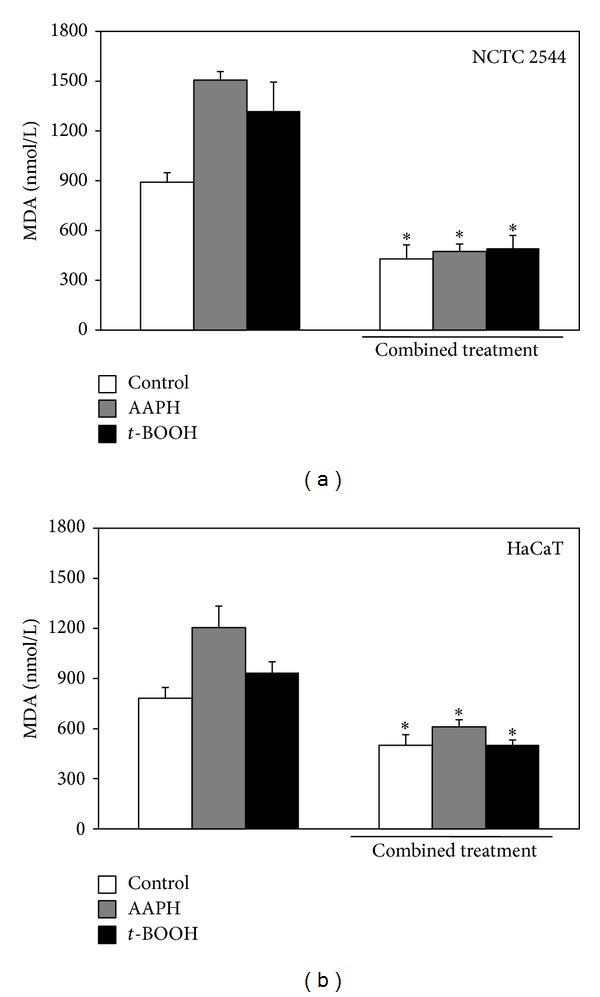
Effect of the combined treatment with all the seven supplement's components on lipid peroxidation in keratinocytes exposed or not to prooxidant agents. Cells were treated simultaneously with all the components for 24 h and then exposed for 1 h to the prooxidant agents AAPH (25 mM) or* t*-BOOH (0.25 mM) or vehicle alone. Each component was used at the highest concentration reported in Section 2. Lipid peroxidation was evaluated spectrophotometrically as MDA production. Data are the means ± SE of a number of determinations ranging from 3 to 6. ∗: significantly different from respective control (NCTC 2455: CT versus control, *P* < 0.007; CT +AAPH versus AAPH, *P* < 0.0003; CT +*t*-BOOH versus* t-*BOOH, *P* < 0.04; HaCaT: CT versus control, *P* < 0.03; CT +AAPH versus AAPH, *P* < 0.02; CT +*t*-BOOH versus* t-*BOOH, *P* < 0.03, two-tailed unpaired *t*-test).

**Figure 6:**
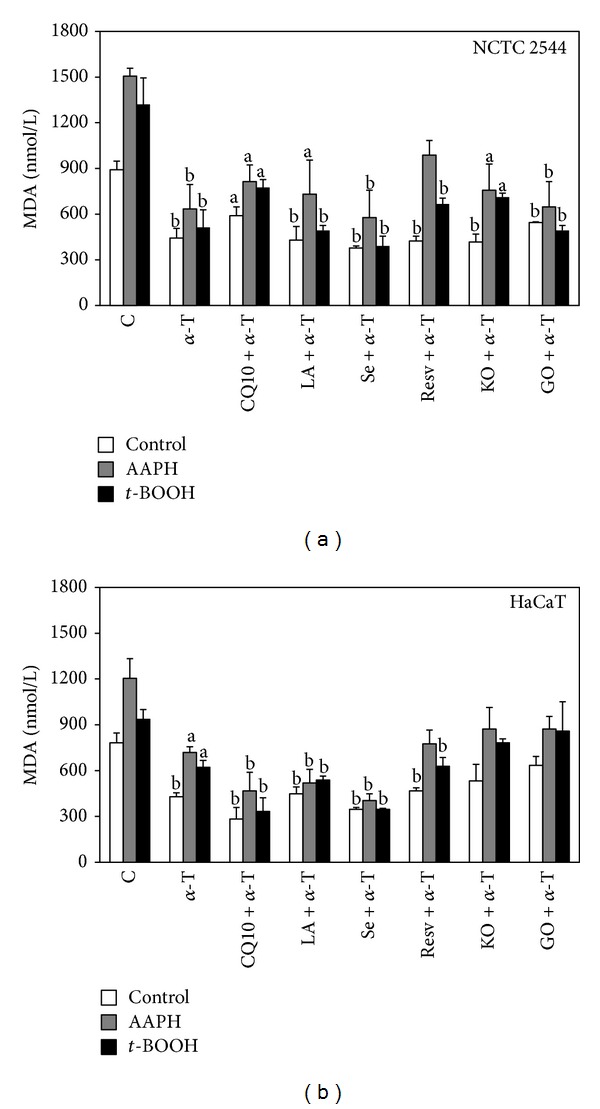
Effect of *α*-T alone or combined with each of the other supplement's components on lipid peroxidation in keratinocytes exposed or not to prooxidant agents. Cells were treated with *α*-T alone or combined with each of the other supplement's components for 24 h and then exposed for 1 h to the prooxidant agents AAPH (25 mM) or* t*-BOOH (0.25 mM) or vehicle alone. Each component was used at the highest concentration reported in Section 2. Lipid peroxidation was evaluated spectrophotometrically as MDA production. Data are the means ± SE of a number of determinations ranging from 3 to 6. a: significantly different from respective control (*P* < 0.05); b: significantly different from respective control (*P* < 0.01), one-way ANOVA followed by Dunnett's test.

**Figure 7:**
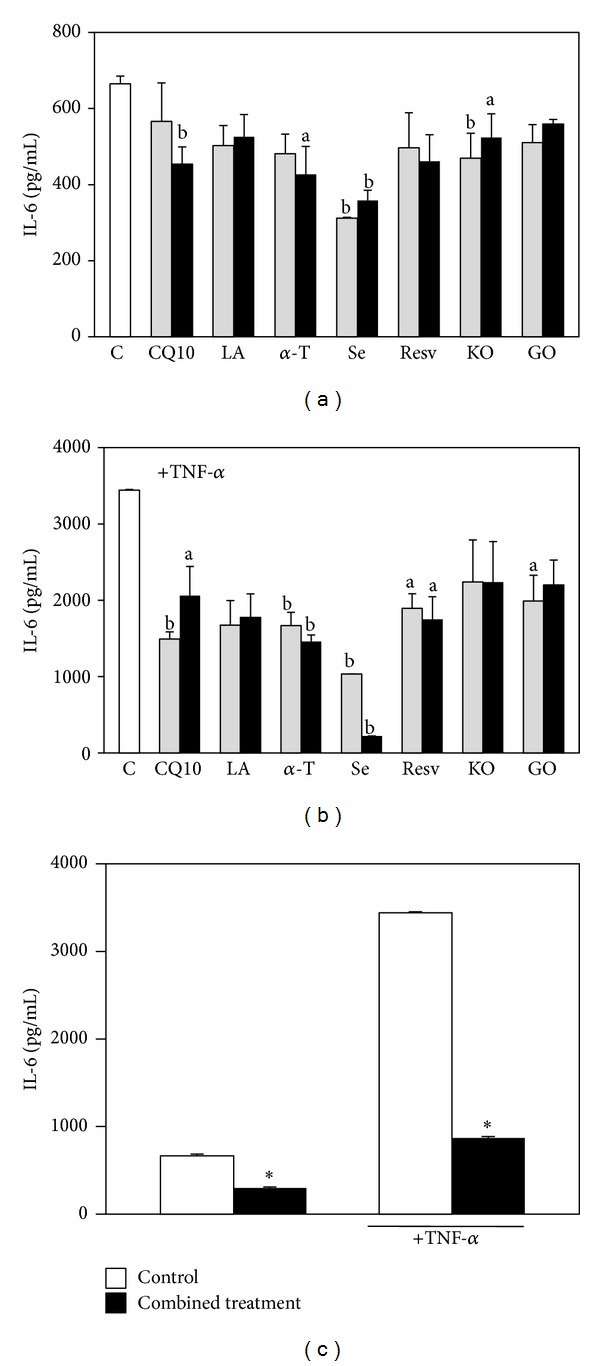
Effect of the seven supplement's components on HaCaT keratinocyte IL-6 production. Cells were exposed for 18 h to increasing concentrations of the components in the absence (a) or in the presence (b) of TNF-*α* (20 ng/mL, 6 h pretreatment and further 18 h with the components); the two concentrations used for each component are the lowest and highest concentrations reported in Section 2. Data are the means ± SE of 4 determinations. a: significantly different from control (*P* < 0.05); b: significantly different from control (*P* < 0.01), one-way ANOVA followed by Dunnett's test. (c) Cells were treated simultaneously with all the components, both in basal conditions or in the presence of TNF-*α* (20 ng/mL), following the timing of the previous experiment. Each component was used at the highest concentration reported in Section 2. Data are the means ± SE of 4 determinations. ∗: significantly different from respective control (*P* < 0.0001, two-tailed unpaired *t*-test).

**Figure 8:**
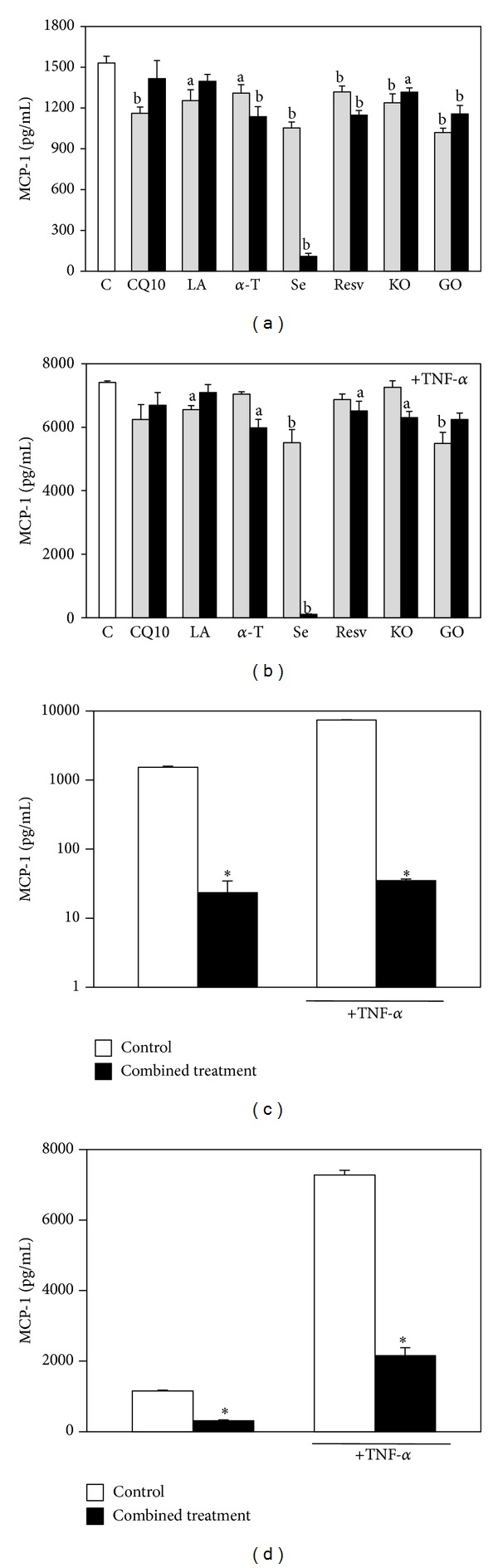
Effect of the seven supplement's components on HaCaT keratinocyte MCP-1 production. Cells were exposed to increasing concentrations of the components in the absence (a) or in the presence (b) of TNF-*α* (20 ng/mL, 6 h pretreatment and further 18 h with the components); the two concentrations used for each component are the lowest and highest concentrations reported in Section 2. Data are the means ± SE of a number of determinations ranging from 4 to 6. a: significantly different from control (*P* < 0.05); b: significantly different from control (*P* < 0.01), one-way ANOVA followed by Dunnett's test. (c) Cells were exposed simultaneously to all the components, both in basal conditions or in the presence of TNF-*α* (20 ng/mL), following the timing of the previous experiment. Each component was used at the highest concentration reported in Section 2. Data are the means ± SE of a number of determinations ranging from 3 to 6. ∗: significantly different from respective control (*P* < 0.0001, two-tailed unpaired *t*-test). (d) The cells were exposed simultaneously to all the components excluding Se, both in basal conditions or in the presence of TNF-*α* (20 ng/mL), following the timing of the previous experiments. Each component was used at the highest concentration reported in Section 2. Data are the means ± SE of a number of determinations ranging from 6 to 8. ∗: significantly different from respective control (*P* < 0.0001, two-tailed unpaired* t*-test).

Data (expressed as the means ± SE) were analyzed by either two-tailed unpaired* t*-test (Figures 1, 4, 5, 7(c), 8(c), and 8(d)) when two groups were compared or by one-way analysis of variance (ANOVA) followed by Dunnett's test when three or more groups were compared (Figures 2, 3, 6, 7(a), 7(b), 8(a), and 8(b)) (InStat GraphPad software).

## 3. Results and Discussion 

In the present work the antioxidant and anti-inflammatory potential of a combined treatment with seven natural compounds/oils (CoQ, LA, Resv, Se, *α*-T, GO, and KO) was evaluated* in vitro*. These compounds/oils are known for their ability to reduce oxidative stress and/or inflammation in keratinocytes and other types of cells (Table 1). The combined treatment reproduces the formulation of a dietary supplement indicated for the prevention of skin pathologies. Two different keratinocyte lines (HaCaT and NCTC 2544) showing different degrees of differentiation [11, 12] were used. In agreement (Figure 1), it was confirmed by immunocytochemistry that whereas 30% of HaCaT cells were positive to cytokeratin-13 (CK-13), a specific marker of keratinocyte differentiation [39], only 10% of NCTC 2544 became positive to CK-13.

### 3.1. Antioxidant Activities of the Compounds/Oils Alone or in Combination

The capacity of each component to inhibit ROS production in the keratinocytes in basal conditions (Figure 2) or in the presence of a prooxidant (100 *μ*M H_2_O_2_) (Figure 3) was first examined. Each component was used within a range of concentrations known to produce antioxidant or other beneficial effects in normal or cancer cells cultured* in vitro* (Table 1). After 24 h of treatment and in basal conditions (Figure 2), all the components analyzed (except for GO in NCTC 2544 cells) significantly reduced ROS production to a comparable extent in both the cell lines (Figure 2). However, when a prooxidant agent (100 *μ*M H_2_O_2_, Figure 3) was added to NCTC 2544 after 24 h, only *α*-T and Se were still able to retain their significant effect at all the concentrations tested (Figure 3(a)), even though with less efficiency. Both *α*-T and Se at the maximal concentrations inhibited ROS production by 86% in basal conditions and by 49% and 61% with H_2_O_2_, respectively. Instead, in HaCaT cells treated with H_2_O_2_ the inhibitory effect on ROS production was still observed (Figure 3(b)) with all the components, except for Resv and KO at the lowest concentrations. However, on the whole, in the presence of H_2_O_2_, a similar maximal inhibition of about 50% was obtained with all the compounds/oils (excluding KO) in both the cell lines. Similarly, in basal conditions the maximal inhibition obtained was similar for all the compounds (excluding GO in NCTC 2544), being in the ranges of 75–90% in NCTC 2544 and 60–90% in HaCaT cells.

The inhibitory effect of a combined treatment with all the compounds/oils on the cellular production of ROS was next analyzed. When the components were administered in combination at the highest concentrations used, they markedly and significantly reduced ROS production in NCTC 2544 cells, both in basal conditions (93% reduction, *P* < 0.027), as well as with H_2_O_2_ (57% reduction, *P* < 0.0099). However, if these effects were compared with those obtained (Figures 2(a) and 3(a)) treating NCTC 2544 cells with *α*-T alone, both in basal conditions, or with H_2_O_2_, no significant difference was observed. Also in HaCaT cells the combined treatment caused a strong and significant reduction of ROS production (86% reduction in basal conditions, *P* < 0.021; 49% with H_2_O_2_, *P* < 0.0001). Moreover, in the presence of H_2_O_2_, the reduction was significantly higher than that (30%, *P* < 0.05) observed with 30 *μ*M *α*-T alone. However, if the results obtained in the two cell lines were compared, the inhibitory effect of the combined treatment was slightly, even if not significantly, more evident in the NCTC 2544 cells (93% versus 86% inhibition in basal conditions and 57% versus 49% in the presence of H_2_O_2_, in NCTC 2544 and in HaCaT cells, respectively) (Figure 4).

The ability of the combined treatment to inhibit lipid peroxidation was also evaluated in NCTC 2544 and HaCaT cells by assessing MDA production (Figure 5), either in basal conditions or after a treatment with two different prooxidant agents with different specificity of action, as follows: AAPH (25 mM) and* t*-BOOH (0.25 mM). The combined treatment induced a strong and significant reduction of MDA production in all the conditions (see legend of Figure 4 for further detail on significance values). Moreover, also in this case, NCTC 2544 cells showed a slightly higher sensitivity than HaCaT cells to the inhibitory effect of the combined treatment. In particular, the combined treatment reduced MDA production by 52% versus 36% in NCTC 2544 and in HaCaT cells, in basal conditions, by 69% versus 49% in the presence of AAPH, and by 63% versus 46% in the presence of* t*-BOOH, respectively. This is an interesting finding, since it has been reported that differentiated keratinocytes show higher level of endogenous antioxidants and are more resistant than the less differentiated cells to prooxidant insults [40, 41]. A supplementation combining the seven components studied could therefore have the potential to efficiently prevent the oxidative injury, even when it affects the less differentiated cells located in the basal layer of epidermis.

Since in the experimental conditions used *α*-T appeared as one of the compounds showing maximal efficiency in inhibiting ROS production (Figures 2 and 3), its ability to inhibit MDA produced in the presence or absence of AAPH or* t*-BOOH (Figure 6) was also evaluated. In all the experimental conditions *α*-T was able to significantly inhibit MDA production with similar efficiencies, both in NCTC 2544 (basal condition, +AAPH, and +*t*-BOOH: 50%, 58%, and 61% inhibition, respectively, *P* < 0.01) and in HaCaT cells (basal condition: 45%, *P* < 0.01; +AAPH and* t*-BOOH: 40% and 34% inhibition, respectively, *P* < 0.05). In some experimental conditions, the combined treatment was significantly more efficient than the individual 30 *μ*M *α*-T treatment, such as in NCTC 2544 cells in the presence of AAPH (combination: 46% inhibition versus *α*-T: 34% inhibition, *P* < 0.05) or in HaCaT cells in the presence of* t*-BOOH (combination: 69% inhibition versus *α*-T: 58% inhibition, *P* < 0.05). None of the remaining compounds/oils used at the maximal concentrations was able to improve significantly the inhibitory effect produced by 30 *μ*M *α*-T on lipid peroxidation (Figure 6), in both the cell lines and all experimental conditions. The same results were obtained also when the cells were treated with the lowest concentrations used (data not shown). Overall, these results confirm that *α*-T is one of the most powerful natural dietary antioxidants and, if it is assumed that it remains unaltered before ingestion and is efficiently absorbed, its presence in a supplement with other antioxidants may ensure the maximum antioxidant effect.

### 3.2. Anti-Inflammatory Activities of the Compounds/Oils Alone or in Combination

The anti-inflammatory potential of the components, administered alone or in combination with HaCaT cells treated or not with the proinflammatory cytokine TNF-*α* (Figures 7 and 8), was next examined and, in particular, their effect on the production of the proinflammatory cytokines MCP-1, IL-6, and interleukin-1*β* (IL-1*β*) was evaluated. The levels of IL-1*β* secreted by HaCaT cells were too scarce to allow a correct evaluation and its variations were never significant as compared to the control (data not shown). On the other hand, the cells produced high levels of IL-6 and MCP-1, especially when they were stimulated with TNF-*α*. Moreover, it was observed (Figure 7) that all the compounds/oils (excluding LA) evaluated individually were able to significantly inhibit IL-6 secretion in our experimental conditions, even though only at some concentrations and in some of the experimental conditions used (see the significances in the legend of Figure 7). Similarly, MCP-1 production was significantly inhibited by all the compounds (Figure 8; see significance in the legend of Figure 8). The most efficient inhibitor of both IL-6 and MCP-1 production was Se, especially in cells stimulated with TNF-*α* (0.4 *μ*M Se: 93.6% inhibition of IL-6 production, *P* < 0.01; 99.0% inhibition of MCP-1 production, *P* < 0.0001). The combined treatment with all the components (Figures 7(c) and 8(c)) reduced markedly and significantly the production of both IL-6 and MCP-1 in all the experimental conditions studied. In particular, IL-6 production was reduced by 55.0% and 75.0% in basal conditions and after stimulation with TNF-*α*, respectively (*P* < 0.0001) (Figure 7(c)). In the same conditions, the treatment reduced MCP-1 production by 98.0% and 99.5%, respectively (*P* < 0.0001) (Figure 8(c)), and this effect was slightly but significantly higher (*P* < 0.05) than that observed with 0.4 *μ*M Se administered alone, both in the presence and in the absence of TNF-*α*. This finding is interesting, since MCP-1 is one of the cytokines most expressed by keratinocytes during inflammation. It should be emphasized, however, that the combination of compounds/oils cannot inhibit MCP-1 production much more efficiently than Se, given the extremely high inhibitory effect exerted by this compound alone. Se, however, is present in the supplement studied in the inorganic form of Na-selenite, which shows an extremely low absorbance index in humans [42, 43]. For this reason, it may hardly contribute to the expected beneficial effect of the dietary supplement* in vivo*. Nevertheless, it was observed that if the combined treatment was performed excluding Se, it was still able to significantly decrease MCP-1 production by HaCaT cells both in basal conditions and in the presence of TNF-*α* (Figure 8(d)), even though to a lower extent (73.5% and 71.6%, respectively). Interestingly, this inhibition was significantly higher (*P* < 0.001) than that observed exposing the cells to each of its six other components, either in the presence (Figure 8(a)) or in the absence of TNF-*α* (Figure 8(b)).

**Figure 9:**
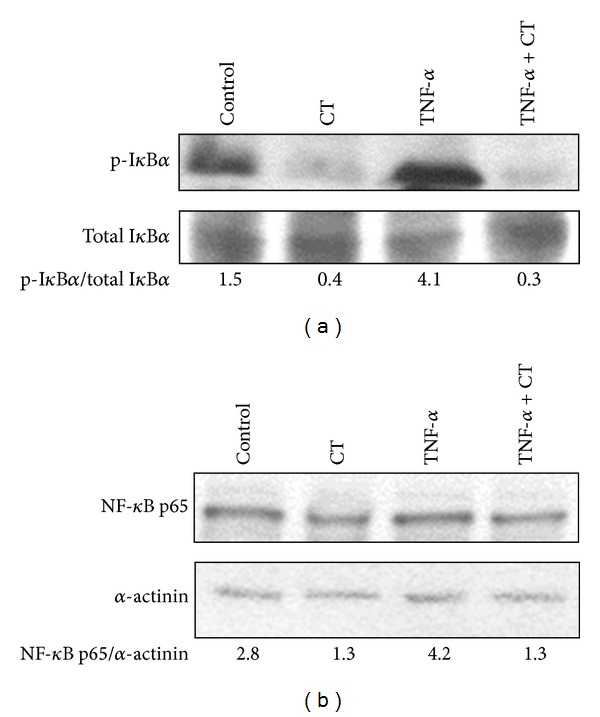
Effect of the combined treatment with all the seven supplement's components on I*κ*B*α* phosphorylation and p65 expression in HaCaT keratinocytes. Cells were exposed to the combined treatment (CT) in the absence or in the presence of TNF-*α* (20 ng/mL) for 5 h. (a) A representative Western Blot of three similar experiments with the corresponding p-I*κ*B*α*/I*κ*B*α* ratio is shown. (b) A representative Western Blot of three similar experiments with the corresponding p65/*α*-actinin ratio is shown.

Afterwards, it was investigated if the combination could regulate the Nuclear Factor-*κ*B (NF-*κ*B) pathway, known to be involved in the synthesis of proinflammatory cytokines (Figure 9). In resting conditions, NF-*κ*B is bound to its inhibitor I*κ*B*α* in a nonphosphorylated form in the cytosol. When, following an inflammatory stimulus such as TNF-*α*, I*κ*B*α* is phosphorylated and then degraded, NF-*κ*B can translocate into the nucleus and activate the transcription of genes encoding proinflammatory cytokines. In agreement, the phosphorylation of I*κ*B*α* (p-I*κ*B*α*) increased in HaCaT cells treated with TNF-*α*. In line with the ability of the combined treatment to inhibit the production of IL-6 and MCP-1, it reduced the phosphorylation of I*κ*B*α* (Figure 9(a)) in HaCaT cells, both in the presence and in the absence of TNF-*α*. Moreover, in the same conditions, it inhibited also the expression of the NF-*κ*B subunit p65, decreasing the amount of NF-*κ*B available for cytokine transcription.

## 4. Conclusions

On the whole, the findings show that the combined treatment of keratinocytes with the antioxidant/anti-inflammatory compounds/oils studied possesses high antioxidant activity that, in specific experimental conditions, was higher than that observed with the individual administration of the most powerful components. The combined treatment showed also powerful anti-inflammatory activities. Particularly, it inhibited more efficiently than all the individual components the secretion of the proinflammatory cytokine MCP-1, which is known to attract monocytes in the skin, where it is expressed at high levels and plays a crucial role in the development of chronic inflammation. In keeping with this finding, the combination inhibited efficiently the molecular pathway associated with NF-*κ*B, the main transcription factor involved in the cellular production of proinflammatory cytokines. On these bases, it can be hypothesized that the simultaneous presence of the antioxidant/anti-inflammatory components in the formulation of a dietary supplement could ensure the achievement of these beneficial effects also* in vivo*. Moreover, the development of a formulation for topical use containing the combination of compounds/oils described could also be advisable. In fact, this route of administration could overcome all the problems related to biodistribution and metabolic conversion and directly target keratinocytes.

## Figures and Tables

**Table 1:** Compound concentrations reported to inhibit oxidative stress or inflammation or to exert other beneficial effects∗ in keratinocytes or other cultured cells.

Compounds	Concentration ranges	Cultured cells	Reference
Coenzyme Q10 (CQ10)	0.5–10 *μ*M	Keratinocytes fibroblasts	[19, 20]
Krill oil (KO)	2.5–20.0 *μ*g/mL	Colon cancer cells	[21, 22]∗∗
Lipoic acid (LA)	0.01–1 mM	Keratinocytes, neurons, muscle cells, and bronchial epithelial cells	[23–26]
Resveratrol (Resv)	0.5–100 *μ*M	Keratinocytes	[27–30]
Na-selenite (Se)	0.1–1 *μ*M	Keratinocytes	[31–33]
*α*-Tocopherol (*α*-T)	10–580 *μ*M	Keratinocytes	[34–38]

∗Antiproliferative effects.

∗∗This reference regards other kinds of marine oil (algal oil or fish oil).
